# Time to optimal glycaemic control and prognostic factors among type 2 diabetes mellitus patients in public teaching hospitals in Addis Ababa, Ethiopia

**DOI:** 10.1371/journal.pone.0220309

**Published:** 2019-07-31

**Authors:** Tigist W. Leulseged, Birhanu T. Ayele

**Affiliations:** 1 Department of Internal medicine, St. Paul’s Hospital Millennium Medical College, Addis Ababa, Ethiopia; 2 Divison of Epidemiology and Biostatistics, Faculty of Medicine and Health Sciences, Stellenbosch University, Cape Town, South Africa; University of Nebraska Medical Center, UNITED STATES

## Abstract

**Aim:**

To estimate time to first optimal glycaemic control and identify prognostic factors among type 2 diabetes mellitus (T2DM) patients attending diabetes clinic of public teaching hospitals in Addis Ababa, Ethiopia.

**Methods:**

A retrospective chart review study was conducted at diabetes clinic of Addis Ababa’s public teaching hospitals among a randomly selected sample of 685 charts of patients with T2DMwho were on follow up from January 1, 2013 to June 30, 2017. Data was collected using data abstraction tool. Descriptive statistics, Kaplan Meier plots, median survival time, Log-rank test and Cox proportional hazard survival models were used for analysis.

**Results:**

Median time to first optimal glycaemic control among the study population was 9.5 months. Factors that affect time to first optimal glycaemic control were age group (HR = 0.635, 95% CI: 0.486–0.831 for 50–59 years, HR = 0.558, 95% CI: 0.403–0.771for 60–69 years and HR = 0.495, 95% CI: 0.310–0.790 for > = 70 years), diabetes neuropathy (HR = 0.502, 95% CI: 0.375–0.672), more than one complication (HR = 0.381, 95% CI: 0.177–0.816), hypertension (HR = 0.611, 95% CI: 0.486–0.769), dyslipidemia (HR = 0.609, 95% CI: 0.450–0.824), cardiovascular disease (HR = 0.670, 95% CI: 0.458–0.979) and hospital patient being treated (HR = 1.273, 95% CI: 1.052–1.541).

**Conclusions:**

Median time to first optimal glycaemic control among T2DM patients is longer than expected which might imply that patients are being exposed to more risk of complication and death.

## Introduction

Diabetes is a chronic, progressive disease characterized by elevated levels of blood glucose. There are three types of diabetes: Type 1, Type2 and gestational diabetes. Type 2 diabetes is the commonest type[1, 2].

Diabetes is one of the largest global health emergencies of the 21^st^ century. Each year more and more people live with this condition and this increase is noted more rapidly in resource limited countries. According to IDF Atlas and WHO, about 45.1% of all adults aged 20–79 years with diabetes in Africa live in four countries including Ethiopia. In Ethiopia, prevalence of diabetes in adults has increased from 2.9% in 2015 to 3.8% in 2016 to 5.2% in 2017[3–5].

People with diabetes can live longer and have a healthy life if their diabetes is detected early and well-managed, with integrated self-management and health professional support. The longer a person lives with undiagnosed, untreated and/or uncontrolled diabetes, the worse their health outcomes are likely to be. Therefore, controlling blood glucose is key in preventing and slowing the progression of complications [1–4].

Studies conducted in different regions of Ethiopia have focused mainly on level of glycaemic control at one point in time. Majority of the studies show poor glycaemic control (60–80% patients in each study have poor glycaemic control) [6–9]. This is also the case in other countries including Uganda, Kenya, India and China [10–13]. Similarly, a systematic review of literatures from 2011–2015 shows that glycaemic control is suboptimal in majority (typically 40%-60%) of people with diabetes in both low- and higher-income countries [14].

In addition, studies conducted among T2DM patients who has been followed over a period of time has shown that old age, being overweight, long-standing diabetes, high HgA1c, LDL-to-HDL cholesterol ratios, hypertension, micro-albuminuria, and previous cardiovascular disease, are important predictors of poor glycaemic control, morbidity and mortality [12, 15–17].

Though knowing the level of glycaemic control of a patient is an important predictor of development of complication and risk of death from diabetes, the other most important predictor which is the time that the patient stayed in that poor glycaemic level before reaching optimal glycaemic control has not been studied yet.

Patients with same level of poor glycaemic control can have different prognosis because of the difference in the time the patients stayed in that poor glycaemic state. The risk of complication and death increases as the patient stays longer in poor glycaemic level.

The objective of this study was to estimate time to first optimal glycaemic control and identify prognostic factors among T2DM patients attending diabetes clinic of public teaching hospitals in Addis Ababa, Ethiopia.

## Methods and materials

### 2.1 Study design and subjects

The study design was hospital-based retrospective chart review and was conducted at two public teaching hospitals in Addis Ababa: St Paul Hospital Millennium Medical College (SPHMMC) and Yekatit 12 Hospital Medical College (Y12HMC). Both hospitals have a diabetes clinic which is under the department of internal medicine. At Y12HMC patients are seen mainly by General Practitioners (GP) and internists. There was no endocrinologist in the hospital during the study period. At SPHMMC patients are seen by internal medicine residents, internists and endocrinologist. The hospitals do not use the national diabetes management intake and follow up guideline consistently. Medical records and information sheets of new T2DM patients’ who were on follow up from 1^st^January, 2013 to 31^st^December, 2017 was reviewed.

### 2.2 Source and Study population

The source population was all new T2DM patients who were on follow up at diabetes clinic of the two hospitals from January 1, 2013 to June 30, 2017. During this interval a total of 1,508 new patients were seen at the two hospitals: 923 patients at SPHMMC and 585 patients at Y12HMC.

The study population was all selected new T2DM patients who full fill the inclusion criteria of >18 years and non-pregnant.

### 2.3 Sample size and sampling procedure

Sample size was determined by using sample size calculation formula forsurvival analysis by considering the following statistical assumptions: 95% Confidence Interval (CI), power of 90%, survival probability of 0.5, 5% marginal error, and loss of 20%. The final sample size for this study was 783.

The estimated total sample size was proportionally allocated to the two study sites according to the number of eligible participants in each site. Finally, cards of 417 from SPHMMC and 269 from Y12HMC that fulfilled the criteria were randomly selected and reviewed.

### 2.4 Operational definitions

**Optimal glycaemic control.** Optimal glycaemic control is defined as the three consecutive month average fasting blood glucose of 80–130 mg/dl with more or less stringent glycemic goals for individual patients based on age/ life expectancy, comorbid conditions, advanced microvascular complications, hypoglycemia unawareness, and individual patient considerations[18]. N.B: FBS is used as a follow up tool in our set up because HgA1c is not consistently available for patient diagnosis and follow up.

**Event.** achieving first optimal glycaemic control.

**Censoring.** patients died, lost to follow-up, transferred out and completed the follow-up period without achieving optimal glycaemic control.

**Time to event.** time between diagnosis up to achieving first optimal glycaemic control or censoring (in month).

- **Start date of the study:**1^st^January, 2013
- **End date of the study:**31^st^December, 2017

### 2.5 Data collection

Pre-tested data abstraction tool (questionnaire) that consists of questions to assess the relevant variables was used to collect the necessary data from the patient medical chart by trained data collectors.

### 2.6 Statistical analysis

The collected data was coded and entered into Epi-Info version 7.2.1.0, cleaned, stored and exported into SPSS version 23 for analysis. Descriptive statistics was presented with frequency tables, Kaplan Meier (KM) plots and median survival times. Kaplan-Meier technique was used to assess survival experience of different groups of patients by using survival curves. Log-rank test was used to assess significant difference among survival distributions of groups for equality.

Univariate analysis was performed to calculate an unadjusted hazard ratio (HR) and to screen out potentially significant independent variables at 25% level of significance. Association between the significant independent variables and the time to first optimal glycaemic control was assessed using multivariable Cox Proportional Hazard (PH) model. Adjusted hazard ratio (HR), P-value and 95% CI for HR were used to test significance and interpretation of results. Variables with p-value ≤ 0.05 were considered as statistically associated with the time to first optimal glycaemic control in months.

### 2.7 Ethical considerations

The study was conducted after obtaining ethical clearance from GAMBY Medical and Business College IRB, Addis Ababa Health Bureau, Y12HMC and SPHMMC. Written informed consent was obtained from Endocrinology or Internal medicine department of the hospitals on behalf of the patients. The study had no any risk/negative consequence for study participants. Medical record number was used for the data collection and personal identifiers of the patient were not used in the research report. Access to the collected information was limited to the principal investigator and confidentiality was maintained throughout the project.

## Result

### 3.1 Censoring status

Among the 685 patients, 483 (70.5%) of the patients achieved optimal glycaemic control while 202 (29.5%) were censored. The median time to achieving optimal glycaemic control was 9.5 months.

### 3.2 Socio-demographic and institution related variables and censoring status

Majority of the patients (27.7%) were in the age range of 50–59 years, 54.6% of the patients were females and majority of the patients were from Addis Ababa (73.7%). More than half (60.9%) of the patients were from SPHMMC and the rest (39.1%) were from Y12HMC.

Higher proportion of patients in the age group 30–39 achieved optimal glycaemic control, followed by 40–49, 50–59, 60–69 and > = 70 years age groups.

The proportion of patients who achieved optimal glycaemic control among females (73.5%) is higher than males (66.9%). Almost seventy percent (69.9%) of patients from Addis Ababa has achieved optimal glycaemic control. The proportion of patients who achieved optimal glycaemic control at SPHMMC (74.1%) is higher than Y12HMC (64.9%). (**Table 1**)

**Table 1:** Socio–demographic and institution related variables and censoring status among T2DM patients, Addis Ababa, 2018 (n = 685).

Variable	Category	Censoring status		Total (%)
		No censored (%)	No of event (%)	
**Age group in years**	30–39	24 (15.6%)	130 (84.4%)	154 (22.5%)
	40–49	36 (22.1%)	127 (77.9%)	163 (23.8%)
	50–59	62 (32.6%)	128 (67.4%)	190 (27.7%)
	60–69	50 (41.0%)	72 (59.0%)	122 (17.8%)
	> = 70	30 (53.6%)	26 (46.4%)	56 (8.2%)
**Sex**	Female	99 (26.5%)	275 (73.5%)	374 (54.6%)
	Male	103 (33.1%)	208 (66.9%)	311 (45.4%)
**Region**	Addis Ababa	152 (30.1%)	353 (69.9%)	505 (73.7%)
	Outside Addis Ababa	50 (27.8%)	130 (72.2%)	180 (26.3%)
**Hospital**	Yekatit 12	94 (35.1%)	174 (64.9%)	268 (39.1%)
	St Paul	108 (25.9%)	309 (74.1%)	417 (60.9%)

### 3.3 Diabetes related variables (diabetes related complications, diabetes hospitalization and medication) and censoring status

Regarding history of diabetes related complication in general, 32.0% of the patients had history of one or more complications. Majority of the patients had neuropathy (16.9%) followed by acute complication (12.7%), nephropathy (5.1%) and other complication (3.8%). Eighty seven (12.6%) patients had diabetes related hospitalization which was mainly due to acute complication. Oral anti-diabetic drug was given to the majority of patients at the initiation of treatment (83.5%) compared to insulin (16.5%).

The proportion of patients who achieved optimal glycaemic control is higher among those with no history of diabetes related complication (75.8%) compared to those with one or more complication (59.4%).

The proportion of patients who achieved the event is lower among those with more than one diabetes related complication (22.0%) compared to those with only one complication or no complication (73.6%).

The proportion of patients who achieved optimal glycaemic control is comparable among those who were on oral anti-diabetic drug and insulin at the time of initiation of treatment (70.3% Vs 71.7%). (**Table 2**)

**Table 2:** Diabetes related variables and censoring status among T2DM patients, Addis Ababa, 2018 (n = 685).

Variable	Category	Censoring status		Total (%)
		No censored (%)	No of event (%)	
**History of diabetes related complication**	No	113 (24.2%)	353 (75.8%)	466 (68.0%)
	Yes	89 (40.6%)	130 (59.4%)	219 (32.0%)
**Acute complication**	No	173 (28.9%)	425 (71.1%)	598 (87.3%)
	Yes	29 (33.3%)	58 (66.7%)	87 (12.7%)
**Diabetes nephropathy**	No	176 (27.1%)	474 (72.9%)	650 (94.9%)
	Yes	26 (74.3%)	9 (25.7%)	35 (5.1%)
**Diabetes neuropathy**	No	145 (25.5%)	424 (74.5%)	569 (83.1%)
	Yes	57 (49.1%)	59 (50.9%)	116 (16.9%)
Other complication*	No	189 (28.7%)	470 (71.3%)	659 (96.2%)
	Yes	13 (50.0%)	13 (50.0%)	26 (3.8%)
**More than one complication**	No	170 (26.4%)	474 (73.6%)	644 (94.0%)
	Yes	32 (78.0%)	9 (22.0%)	41 (6.0%)
**Diabetes related hospitalization**	No	170 (28.4%)	429 (71.6%)	599 (87.4%)
	Yes	32 (37.2%)	54 (62.8%)	86 (12.6%)
**Regimen**	Oral	170 (29.7%)	402 (70.3%)	572 (83.5%)
	Insulin	32 (28.3%)	81 (71.7%)	113 (16.5%)

*Other complication includes diabetes retinopathy, diabetic foot ulcer and diabetes gastropathy

### 3.4 Co- morbid illness and censoring status

More than half (58.8%) of the patients had history of co-morbid illness and 41.2% did not have. Majority of the patients had hypertension (48.6%) followed by dyslipidemia (22.6%), cardiovascular disease (13.9%) and other co-morbid illness (11.1%). More than one fourth of patients (27.6%) had more than one co morbid illness.

The proportion of patients who achieved optimal glycaemic control is higher among those with no history of co-morbid illness (80.9%) than those with one or more co-morbid illness (63.3%). (**Table 3**)

**Table 3:** Co-morbid illness and censoring status among T2DM patients, Addis Ababa, 2018 (n = 685).

Variable	Category	Censoring status		Total (%)
		No censored (%)	No of event (%)	
**History of co-morbid illness**	No	54 (19.1%)	228 (80.9%)	282 (41.2%)
	Yes	148 (36.7%)	255 (63.3%)	403 (58.8%)
**Hypertension**	No	75 (21.3%)	277 (78.7%)	352 (51.4%)
	Yes	127 (38.1%)	206 (61.9%)	333 (48.6%)
**Dyslipidemia**	No	135 (25.5%)	395 (74.5%)	530 (77.4%)
	Yes	67 (43.2%)	88 (56.8%)	155 (22.6%)
**Cardiovascular disease**	No	152 (25.8%)	438 (74.2%)	590 (86.1%)
	Yes	50 (52.6%)	45 (47.4%)	95 (13.9%)
Other co-morbid illness*	No	175 (28.7%)	434(71.3%)	609 (88.9%)
	Yes	27 (35.5%)	49 (64.5%)	76 (11.1%)
**More than one co morbid illness**	No	114 (23.0%)	382 (77.0%)	496 (72.4%)
	Yes	88 (46.6%)	101 (53.4%)	189 (27.6%)

Others*: includes renal disease, neurologic disease, chronic respiratory diseases and thyroid metabolism disorders

### 3.5 Comparison of survival experience

**Fig 1:**
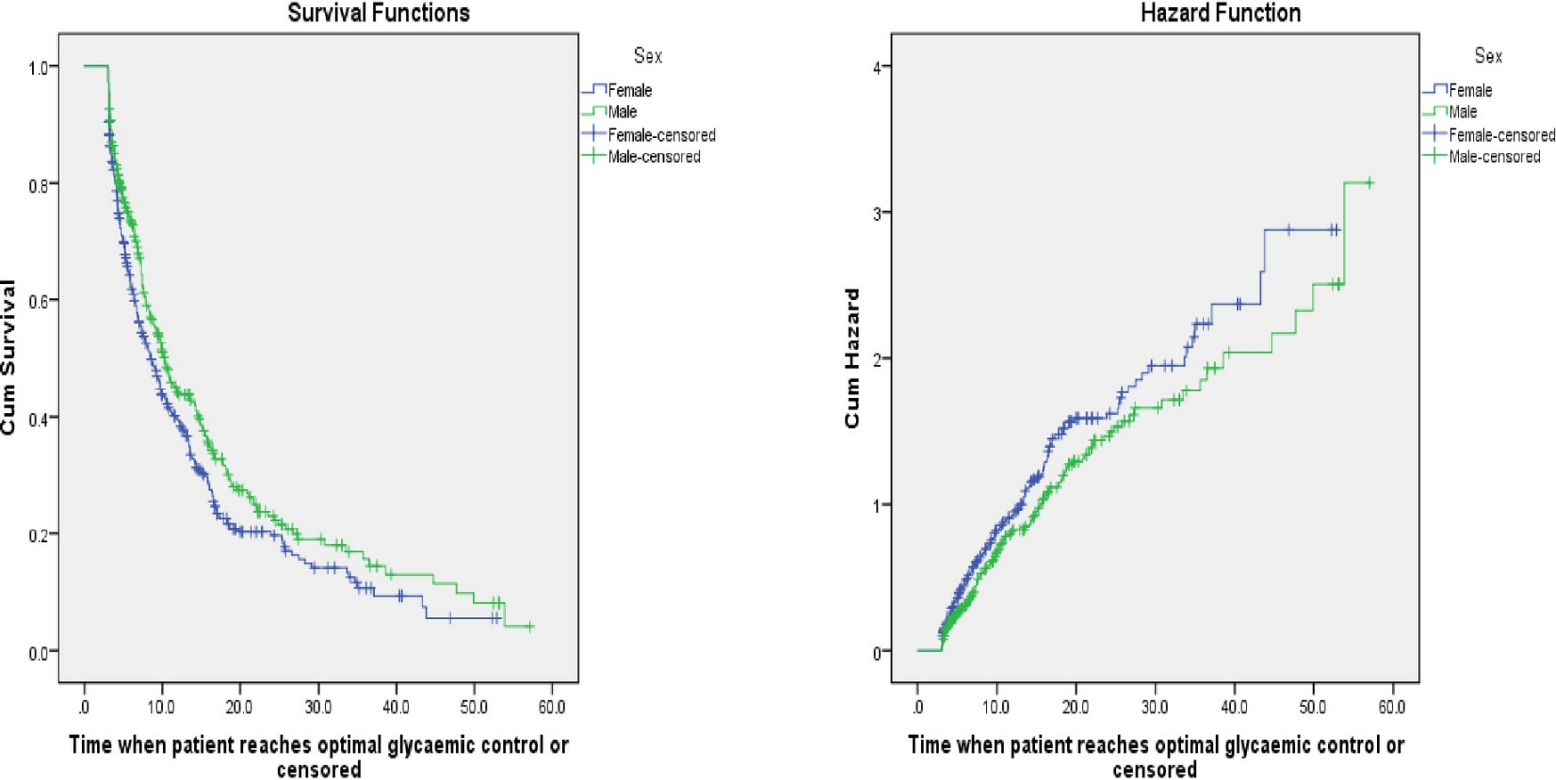
Survival and hazard functions of sex by time, Addis Ababa, 2018.

A log rank test was used to assess difference in the survival distribution among groups. The median survival time showed that females achieved optimal glycaemic control in a relatively shorter time (8.6 months) than males (10.3 months). The log rank test was statistically significant (X^2^_(1)_ = 5.546, P-value = 0.019). As shown in Fig 1 the KM survival function graph also showed that females have a favorable survival (time to achievement of first optimal glycaemic control) experience. The figure shows that, the instantaneous chance of achieving optimal glycaemic control increases for both sexes as the duration of treatment increases.

Regarding age, patients in the age group of 30–39 years showed shorter median time to achieving optimal glycaemic control (5.2 months) followed by patients in the age group 40–49 years (6.5 months). Older patients needed much longer time to achieve optimal glycaemic control; 10.8 months for 50–59 years, 14.8 months for 60–69 years and 28.6 months for > = 70 yearsof age. The survival time was significantly different among the five age groups (X^2^_(4)_ = 129.010, P-value = 0.000).

Having complications seems to extend time to achieve optimal glycaemic control. The average time to achieve optimal glycaemic control was longer among patients with nephropathy (36.5 months) followed by patients with neuropathy (21.7 months) and other complication (18.7 months) and all show statistically significant (all p-values <0.05) difference when compared to the average time of patients with no such complications.

The median time to achieving optimal glycaemic control was longer among patients with cardiovascular disease (20.9 months), those with more than one co-morbid illness (18.4 months), dyslipidemia (16.6 months), other co-morbid illness (16.0 months) and hypertension (14.8 months) and all show statistically significant (all p-values<0.05) difference when compared to the median time of patients with no such complications.

The median time to achieving optimal glycaemic control among patients who has been hospitalized is longer (10.7 months) than those who has not been hospitalized (9.7 months) and it was statistically significant (X^2^_(1)_ = 3.947, P-value = 0.047). (**Table 4)**

**Table 4:** Comparison of optimal glycaemic control among T2DM patients, Addis Ababa, 2018 (n = 685).

Variable	Category	Test of equality over groups				
		Median survival time(months)	Mean survival time(months)	Log rank (mantel cox)		
				Chi square	Df	Pr>chi square
**Age group in years**	30–39	5.2	7.8	129.010	4	0.000
	40–49	6.5	12.4			
	50–59	10.8	16.8			
	60–69	14.8	21.3			
	> = 70	17.0	28.6			
**Sex**	Female	8.6	14.3	5.546	1	0.019
	Male	10.3	17.2			
**Region**	Addis Ababa	9.9	16.1	1.241	1	0.265
	Outside Addis Ababa	8.3	14.1			
**Hospital patient being treated**	Yekatit 12	10.3	17.6	2.988	1	0.084
	St. Paul	8.7	14.4			
**Diabetes related acute complication**	No	9.5	15.3	0.140	1	0.708
	Yes	9.5	18.3			
**Diabetes nephropathy**	No	8.9	14.6	29.448	1	0.000
	Yes	36.5	35.3			
**Diabetes neuropathy**	No	7.8	13.0	58.926	1	0.000
	Yes	21.7	27.3			
**Other complication**	No	9.0	15.1	11.515	1	0.001
	Yes	18.7	27.8			
**Hypertension**	No	5.7	10.5	110.599	1	0.000
	Yes	14.8	20.9			
**Dyslipidemia**	No	7.6	13.5	45.642	1	0.000
	Yes	16.6	23.0			
**Cardiovascular disease**	No	7.9	13.7	44.292	1	0.000
	Yes	20.9	27.3			
**Other co-morbid illness**	No	8.5	14.8	14.475	1	0.000
	Yes	16.0	22.0			
**More than one co-morbid illness**	No	6.9	11.4	105.090	1	0.000
	Yes	18.4	25.9			
**Diabetes related hospitalization**	No	9.4	14.7	3.947	1	0.047
	Yes	10.7	20.9			
**Regimen**	Oral	9.7	15.2	0.009	1	0.924
	Insulin	8.7	16.3			

### 3.6 Results of multivariable cox proportional hazard model

**Fig 2:**
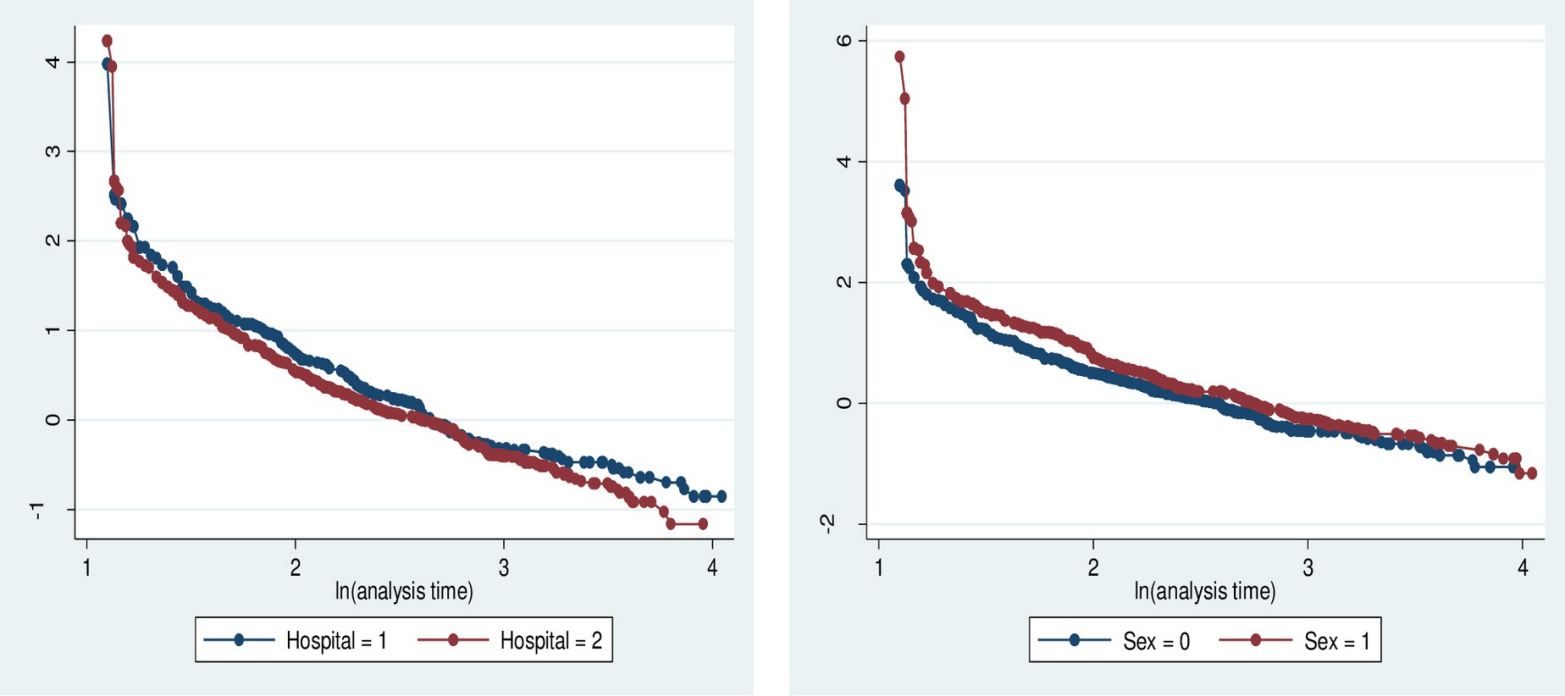
Log minus Log function for hospital and sex, 2018, Addis Ababa.

The fundamental assumption of Cox Proportional Hazard model, proportional hazards assumption, was tested using Log minus Log function on STATA version 14. Parallel lines between groups indicate proportionality [19]. Fig 2 reveals that the survival curves seem parallel throughout the study time. These plots show reasonable fit to the proportional hazard assumption.

From univariate analysis of the independent variables at 25% level of significance; sex, age group, diabetes neuropathy, other complication, more than one complication, hypertension, dyslipidemia, cardiovascular disease, other co-morbid illness, more than one co-morbid illness, diabetes related hospitalization and hospital patient being treated were significantly associated with time to optimal glycaemic control among T2DM patients.

However; only age group, diabetes neuropathy, more than one complication, hypertension, dyslipidemia, cardiovascular disease and hospital patient being treated were found to be significantly associated with time to optimal glycaemic control in the multivariable Cox proportional hazard model at 5% level of significance.

The presence of interaction among the independent variables was checked but there was no significant interaction.

Accordingly, after adjusting for other covariates, compared to those in the age range of 30–39 years, the rate of achieving optimal glycaemic control among those in the age group 50–59, 60–69 and > = 70 years were lower by 36.5%, 44.2% and 50.5%, respectively.

The rate of achieving optimal glycaemic control among patients with neuropathy was lower by 49.8% compared to patients with no neuropathy (HR = 0.502, 95% CI = 0.375–0.672, p-value = 0.000). This means, the time needed to reach optimal glycaemic control among patients with no neuropathy was significantly shorter compared with patients with neuropathy.

Similarly, the rate of achieving optimal glycaemic control among patients with more than one complication was 62% lower than patients with no or one complication (HR = 0.381, 95% CI = 0.177–0.816, p-value = 0.013).

Regarding presence of co-morbid illness, after adjusting for other covariates, the rate of achieving optimal glycaemic control among patients with hypertension, dyslipidemia and cardiovascular disease were respectively lower by 38.9%, 39.1% and 33.0% compared to patients with no hypertension, dyslipidemia and cardiovascular disease.

The rate of achieving optimal glycaemic control among patients treated at SPHMMC is 1.273 times patients treated at Y12HMC (HR = 1.273, 95% CI = 1.052–1.541, p-value = 0.013). (**Table 5**)

**Table 5:** Results for the final Cox proportional hazard model among T2DM patients,Addis Ababa, 2018(n = 685).

Variables	HR (Exp(B)	95.0% CI for HR	Sig.
**Age in years**			
30–39 (R)			0.001*
40–49	0.808	(0.627, 1.042)	0.101
50–59	0.635	**(0.486, 0.831)**	**0.001**
60–69	0.558	**(0.403, 0.771)**	**0.000**
> = 70	0.495	**(0.310, 0.790)**	**0.003**
**Sex**			
Female (R)	1		
Male	0.838	(0.698, 1.007)	0.059
**Diabetes neuropathy**			
No (R)	1		
Yes	0.502	**(0.375, 0.672)**	0.000*
**Other complication**			
No (R)	1		
Yes	0.634	(0.340, 1.184)	0.153
**More than one complication**			
No (R)	1		
Yes	0.381	**(0.177, 0.816)**	0.013*
**Hypertension**			
No (R)	1		
Yes	0.611	**(0.486, 0.769)**	0.000*
**Dyslipidemia**			
No (R)	1		
Yes	0.609	**(0.450, 0.824)**	0.001*
**Cardiovascular disease**			
No (R)	1		
Yes	0.670	**(0.458, 0.979)**	0.039*
**Other co-morbid illness**			
No (R)	1		
Yes	0.705	(0.490, 1.014)	0.059
**More than one co-morbid illness**			
No (R)	1		
Yes	0.891	(0.589, 1.347)	0.583
**Hospital patient being treated**			
Yekatit 12 (R)	1		
St Paul	1.273	**(1.052, 1.541)**	0.013*
**Diabetes related hospitalization**			
No (R)	1		
Yes	0.791	(0.587, 1.065)	0.122

**Note:** HR, Hazard ratio; CI, Confidence interval

* Statistically significant

## Discussion

In our study, the median time to achieving optimal glycaemic control was 9.5 months. Though no clear cut off point is set about when to reach optimal glycaemic control, the trend is to have frequent visits and strict follow up for a newly diagnosed T2DM patient till the patient achieves optimal glycaemic control. Therefore, with the measurement tool that we are using for the study, three consecutive months average fasting blood sugar, patients are expected to reach target at the 3^rd^ month or may be a bit longer than that.

Since there is no previous similar study, the results of this study is compared with cross sectional studies on optimal glycaemic control and survival studies with the event of interest being death and diabetes related morbidity and mortality. The identified prognostic factors of this study are found to be analogous with these literatures.

The age of patients is found to be an important factor that determines time to first optimal glycaemic control. The study shows that the time needed to reach first optimal glycaemic control doesn’t show significant difference between 30–39 and 40–49 years of age. This may be due to the fact that relatively younger patients (30–39 and 40–49) have less co-morbid illness that can affect diabetes disease prognosis and there chance of adherence to follow up and treatment is thought to be relatively better. On the other hand, time needed to reach optimal glycaemic control is longer among patients > = 70 years followed by the age group 60–69 and 50–59 years compared to patients in 30–39 years age group indicating that for patients older than 50 years, as age increases the rate of achieving optimal glycaemic control decreases. This finding is in line with studies conducted in Charleston, South Carolina and India[12, 17].

The study found that having diabetes related complication particularly neuropathy and having more than one diabetes related acute or chronic complication is an important prognostic factor. Patients with neuropathy and more than one complication tend to achieve optimal glycaemic control at a rate of 49.8% and 62% lower that of patients with no neuropathy and patients with no or one complication. This is in accordance with a study conducted at Yekatit 12 hospital and Nantong University hospital [10, 15, 20].

In addition, having co-morbid illness is found to be an important prognostic factor that affects time to optimal glycaemic control. The rate of achieving optimal glycaemic control among patients with hypertension, dyslipidemia and cardiovascular disease were lower by 38.9%, 39.1% and 33.0% compared with patients with no such illnesses showing that dyslipidemia has a more negative influence on individual diabetes control followed by hypertension and then cardiovascular disease. This is because having co-morbid illness has effect on diabetes disease progress and could also be due to the effect of taking many drugs which can lead to drug interaction and also decreased drug adherence which interferes with drug effectiveness. This finding is in line with studies conducted in Charleston, South Carolina, Sweden,Yekatit 12 hospital, Arabian Gulf council countries and Kenya [13, 15–17, 21].

Our study also identified hospital where T2DM patients were treated as one of the factors significantly associated with time to first optimal glycaemic control. The rate of achieving optimal glycaemic control among patients at SPHMMC is 27.3% higher than patients at Y12HMC. This difference could be because of difference in underlying population characteristics or unequal sample sizes of the two hospitals, the sample size from SPHMMC was more than 1.5 times the sample size of Y12HMC (268). It could also be due to difference in the level of health professionals who work at the diabetes clinics as having more professional experience and better exposure and training might be an important contributor for better patient management. The other contributing factor could be the non-consistent use of the national diabetes management and follow up guideline. Using the guideline might help the professionals to address all the relevant factors in patient management in a consistent way and might result comparable outcome between the hospitals.

## Conclusion

Median time to first optimal glycaemic control among T2DM patients attending diabetes clinic of Y12HMC and SPHMMC is longer than expected which might imply that patients are being exposed to more risk of complication and death. This increased risk remains higher for these patients even after they achieved optimal glycaemic control compared to those who achieved optimal glycaemic control in a shorter duration.

## Supporting information

S1 Dataset (SAV)

## Abbreviations

CI: Confidence Interval
DM: Diabetes Mellitus
HDL: High density lipoprotein
HgA1c: Glycated hemoglobin
HMIS: Health Management Information System
HR: Hazard ratio
IDF: International Diabetes Federation
IRB: Institution Review Board
KM: Kaplan Meier
LDL: Low density lipoprotein
PH: Proportional Hazard
SPSS: Statistical Product and Service Solutions
T2DM: Type 2 Diabetes mellitus
WHO: World Health Organization
